# Renal Transplantation in Secondary Amyloidosis Associated with Tuberculosis

**DOI:** 10.1155/2013/353529

**Published:** 2013-12-25

**Authors:** Vivek B. Kute, Aruna V. Vanikar, Himanshu V. Patel, Manoj R. Gumber, Pankaj R. Shah, Pranjal R. Modi, Hargovind L. Trivedi

**Affiliations:** ^1^Department of Nephrology and Clinical Transplantation, Institute of Kidney Diseases & Research Center, Dr. H. L. Trivedi Institute of Transplantation Sciences (IKDRC-ITS), Civil Hospital Campus, Asarwa, Ahmedabad, Gujarat 380016, India; ^2^Department of Pathology, Laboratory Medicine, Transfusion Services and Immunohematology, IKDRC-ITS, Ahmedabad, India; ^3^Department of Urology and Transplantation, IKDRC-ITS, Ahmedabad, India

## Abstract

Although end-stage renal disease (ESRD) related to AA amyloidosis nephropathy secondary to tuberculosis is most common in our country, there are limited data concerning patient and graft outcome after renal transplantation (RTx). To the best of our knowledge, this is the first report of RTx in ESRD patient with secondary amyloidosis due to tuberculosis from India. A 30-year-old female with past history of pulmonary tuberculosis 3 years back was admitted with complaint of gradually progressive pedal oedema and nausea for 3 months. Renal biopsy was suggestive of secondary renal amyloidosis with vascular involvement and chronic tubulointerstitial involvement. She was transplanted with kidney from her 28-year-old brother with 3/6 human leukocyte antigen match. She had immediate good graft function without any perioperative complications (cardiovascular, infections, rejection and delayed graft function). She was discharged with serum creatinine of 0.8 mg/dL. Her last serum creatinine level was 0.9 mg/dL with cyclosporine level of 100 mg/dL at 9-month followup without any medical or surgical complication. The quality of life also improved after transplantation. With careful selection, ESRD patients with secondary amyloidosis due to tuberculosis are eligible for RTx with favorable outcome and improved quality of life.

## 1. Introduction

The most frequent and life-threatening complication of secondary amyloidosis (AA) is renal disease characterized by nephrotic syndrome and a progressive decline of renal function leading to end-stage renal disease (ESRD). Kidney involvement constitutes a major prognostic factor in patients with secondary amyloidosis. Chronic inflammatory diseases (rheumatoid arthritis (RA)) and infections (tuberculosis (TB)) were the most common causes of renal amyloidosis in the developed countries and developing countries [1, 2]. Estimated frequency of AA amyloidosis in Western Europe is about 1/100,000. It comprises 2-3% of total amyloidosis. Overall incidence is about 0.5–1.3/100,000 annually. A total of 2401 renal biopsies were analyzed retrospectively from 1990 to 2008 in an Indian single centre, out of which 8% showed amyloidosis [3]. ESRD patients with AA amyloidosis are considered less suitable for renal transplantation (RTx) due to fear of cardiovascular, infectious complications, risk of graft loss from recurrent amyloid/progressive disease, and high risk of mortality [4]. Although ESRD related to AA amyloidosis nephropathy secondary to TB is most common in our country, there are limited data concerning patient and graft outcome after RTx. To the best of our knowledge, this is the first report of RTx in a patient with amyloidosis due to TB from India.

## 2. Case Presentation

A 30-year-old female was admitted with complaint of gradually progressive pedal oedema and nausea for 3 months. There was no history of skin rash, joint pain, hair loss, or decreased urine output. She had completed the treatment for pulmonary tuberculosis (PTB) in 2009 and responded well. She had no history of any other major illness like diabetes and hypertension. There was no other history of any other major illness in past or family. On examination she had pitting pedal oedema with blood pressure of 110/70 mmHg, temperature of 39°C, respiratory rate of 18 breaths per minute, and heart rate of 90 breaths per minute.

## 3. Investigations

**Figure 1:**
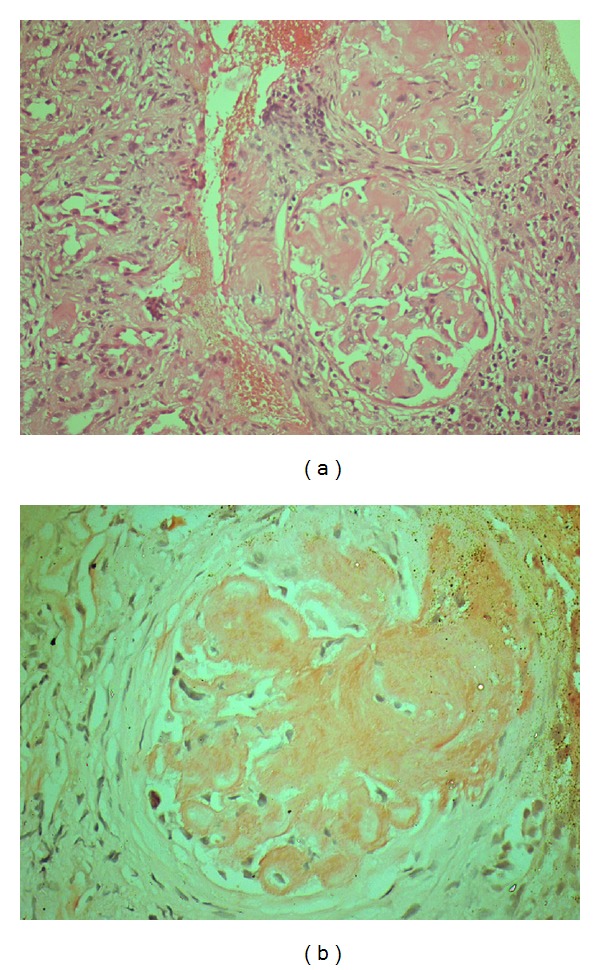
(a) Renal biopsy showing amyloid deposit, pink acellular hyaline material (hematoxylin and eosin stain ×400). (b) Renal biopsy showing congo red positive amyloid deposit, pink acellular hyaline material (congo red stain ×400).

Laboratory investigations revealed the following: hemoglobin, 6.9 gm/L; total white cell count, 11.07 × 103/*μ*L (differential count: 67% neutrophils, 29% lymphocytes, 2% monocytes, and 3% eosinophils); platelet count, 374 × 103/*μ*L; reticulocyte count, 1%; peripheral blood smear was negative for hemolysis; sodium, 145 mmol/L; potassium, 3.8 mEq/L; chloride, 105 mmol/L; urea, 121; creatinine was 7.2 mg/dL; calcium, 7.8 md/dL; phosphate, 8.7; serum protein 5.3 gm/dL. Serum albumin/globulin was 4.3/1.7 gm/dL. Urine examination showed proteinuria (albumin +3) with no hematuria, and pus cells. 24-hour urine protein was 5.3 gm. Enzyme-linked immunosorbent assays for human immunodeficiency virus, hepatitis B surface antigen, and hepatitis C virus were negative with antinuclear antibodies and antibodies to double-stranded DNA negative and normal C3 and C4 levels. Ultrasonography of the abdomen showed kidney size of 12.8 × 4 centimeter on the right side and 11.5 × 4.7 centimeter on the left side. Abdominal fat biopsy was suggestive of amyloidosis. Renal biopsy (Figures 1(a) and 1(b)) was done, suggestive of renal amyloidosis (secondary) with vascular involvement and chronic tubulointerstitial involvement. Blood, urine, and sputum culture did not show any organisms. Electrocardiography was normal. Sputum for acid-fast bacillus was negative. Echocardiogram was normal with ejection fraction of 60%. High resolution computed tomography of chest showed traction bronchiectasis in both lung fields with multiple enlarged lymph nodes in pretracheal, prevascular, and subcarinal and both hilar regions along with hypodense area within nodes suggesting tuberculosis. Erythrocyte sedimentation rate was 30 and 62 mm/hour at end of 1 and 2 hours. Tuberculin test was negative.

## 4. Treatment

Antituberculosis treatment was restarted in July 2012 with isoniazid, rifampicin, pyrazinamide, moxifloxacin, and ethambutol. Pneumococcal polyvalent vaccine was given to prevent infection. She was on maintenance hemodialysis for 4 months through left jugular dual lumen catheter initially for 3 weeks and later through arteriovenous fistula. She was transplanted with kidney from her 28-year-old brother with 3/6 human leukocyte antigen match on the 5th of November 2012. Warm ischaemia time, cold ischaemia time, and anastomosis time were 1 minute and 57 seconds, 52 minutes and 28 minutes and 32 seconds, respectively.

## 5. Immunosuppressive Regimen

Induction immunosuppression consisted of methylprednisolone 500 mg for 3 days. Maintenance immunosuppression consisted of prednisolone 10 mg, cyclosporine 3 mg/kg, and mycophenolate mofetil 500 mg TID. The recipient was covered with perioperative antibiotic. She was initiated on prophylaxis against cytomegalovirus infection (gancyclovir × 3 months), fungal infections (fluconazole × 3 months), and *Pneumocystis carinii* pneumonia (trimethoprim/sulfamethoxazole × 9 months) as per standard protocol.

## 6. Transplantation Outcome and Followup 

She had immediate good graft function without any perioperative surgical or medical complications (cardiovascular, infections, rejection, and delayed graft function). She was discharged with serum creatinine of 0.8 mg/dL. Her last serum creatinine level was 0.9 mg/dL with cyclosporine level of 100 mg/dL at 9-month followup without any complication. Antituberculosis treatment was completed after RTx and continued with isoniazid for TB prophylaxis. The quality of life before and after transplant patients was evaluated using WHOQOL-BREF scale. Physical health, psychological, social relationship, and environment scores before RTx were higher (24; 23; 12; 32) than pretransplant scores (20; 17; 9; 24).

## 7. Discussion

Patients with renal amyloidosis who progress to ESRD can be treated with either dialysis or RTx. Hemodialysis and continuous ambulatory peritoneal dialysis (CAPD) appear to be equally effective, the limiting factors being the degree of extrarenal amyloid deposition, hypotension with hemodialysis, and peritonitis with CAPD. 14% of deaths on dialysis resulted from amyloidosis complications, emphasizing that thorough evaluation of systemic complications remains important even after patients with renal amyloidosis progress to ESRD. Underlying etiology should be corrected or controlled. Successful treatment of the underlying inflammatory process included surgical resection of the focus of infection, cytotoxic agents/biological agents in RA, and antibiotics with chronic infection. RTx should be performed, at earliest time possible after onset of maintenance dialysis prior to development of severe cardiac disease. The outcome appears to be best in patients without cardiac involvement on echocardiography [5, 6].

Fitness of transplant candidates is a major concern due to cardiovascular instability, infections, and uncontrolled underlying inflammatory process. It is important to evaluate the presence, and severity of coronary disease, heart failure, valvular disease, and arrhythmias prior to transplantation. Goals of cardiac testing are to assist in determining transplant candidacy and to identify patients who might benefit from preoperative cardiac intervention and aggressive risk factor modification to decrease perioperative and posttransplant cardiac events [7, 8].

Limited data suggest that graft survival is similar but patient survival is lower in patients with AA amyloidosis compared to other forms of renal disease [9–14]. Recurrence of amyloid nephropathy is of concern because recurrent amyloid deposition in the transplant occurs in 20 to 72% of cases due to continued activity of the underlying disease, but graft loss due to recurrence is uncommon.

Outcomes were sought among all patients attending the UK National Amyloidosis Centre [9] who received RTx between 1978 and 2011. A total of 111 RTx operations were performed in 104 patients. Outcomes following RTx were generally excellent in these diseases, reflecting their slow natural history; median graft survival was 13.1 years [9].

The Australia and New Zealand Dialysis and Transplant Registry reported long-term renal replacement therapy (RRT) outcomes of amyloidosis. The survival of amyloidosis patients receiving peritoneal dialysis was comparable with that of those receiving haemodialysis. Forty-six patients underwent RTx with first graft survival rates of 45% at 5 years and 26% at 10 years. Nine (16.4%) patients experienced amyloidosis recurrence in their allografts, which led to graft failure in six patients. Amyloidosis was associated with poor patient survival following dialysis and/or RTx, poor renal allograft survival, and a significant incidence of disease recurrence in the allograft. An appreciable proportion of amyloid ESRD patients died of amyloidosis-related complications [10].

The French multicenter study [11] reported long-term outcome of RTx in patients with amyloidosis (*n* = 59) compared to the general transplant population (*n* = 177). The overall 5- and 10-year patient survival was significantly lower for the AA amyloidosis patients, but they had similar graft survival. AA amyloidosis transplanted patients exhibited a high proportion of infectious complications after transplantation (73.2%). This study reported that patients with AA amyloidosis nephropathy are eligible for RTx but require careful management of both cardiovascular and infectious complications to reduce the high risk of mortality (73.2%).

Sherif et al. reported similar long-term (5 to 10 years) outcome of live related donor RTx in patients with amyloidosis (*n* = 23) compared to the general transplant population [12]. Emiroglu  et al. reported, 5-year outcome of RTx in patients with amyloidosis (*n* = 32) similar to that in those patients who receive transplantations for other reasons [13]. Better survival was noted for ESRD patients amyloidosis who had RTx despite recurrences due to systemic. These results encourage transplantation in amyloid ESRD [14]. The recurrence of amyloid in the graft within one year after transplantation in a patient with systemic AA amyloidosis secondary to tuberculosis has been reported [15].

It is reasonable to suspect renal amyloidosis in any patient with known history of PTB presenting with pedal edema and proteinuria. The interval between the onset of tuberculosis and first evidence of amyloidosis is variable (months to years). Renal amyloidosis in children within 2 years of the onset of juvenile rheumatoid arthritis at the age of 7 years has been reported. Once amyloidosis has extensively involved the kidneys, anti-TB treatment will not cause any regression in the course of renal amyloidosis. Adequately treated patients may present with renal amyloidosis despite having effective anti-TB therapy. It could be attributed to posttubercular bronchiectasis or an irreversible process of amyloid deposition that had initiated earlier.

In the near future stem cell therapy may have a role in reducing requirement of immunosuppressants leading to less infections and better graft survival.

## 8. Conclusion

With careful selection, patients with secondary amyloidosis due to tuberculosis are eligible for renal transplantation with favorable outcome and improved quality of life.
